# Breaking Therapeutic Inertia in Type 2 Diabetes: Active Detection of In-Patient Cases Allows Improvement of Metabolic Control at Midterm

**DOI:** 10.1155/2015/381415

**Published:** 2015-05-18

**Authors:** Anna M. Lucas Martín, Elena Guanyabens, R. Zavala-Arauco, Joaquín Chamorro, Maria Luisa Granada, Didac Mauricio, Manuel Puig-Domingo

**Affiliations:** ^1^Endocrinology and Nutrition Service, Germans Trias i Pujol Research Institute and Hospital, Department of Medicine, Autonomous University of Barcelona, Can Ruti Campus, Ctra. Canyet s/n, Badalona, 08916 Barcelona, Spain; ^2^Hormone Laboratory, Germans Trias i Pujol Research Institute and Hospital, Department of Medicine, Autonomous University of Barcelona, Can Ruti Campus, Ctra. Canyet s/n, Badalona, 08916 Barcelona, Spain

## Abstract

Type 2 diabetes (T2D) exists in 25–40% of hospitalized patients. Therapeutic inertia is the delay in the intensification of a treatment and it is frequent in T2D. The objectives of this study were to detect patients admitted to surgical wards with hyperglycaemia (HH; fasting glycaemia > 140 mg/dL) as well as those with T2D and suboptimal chronic glycaemic control (SCGC) and to assess the midterm impact of treatment modifications indicated at discharge. A total of 412 HH patients were detected in a period of 18 months; 86.6% (357) had a diagnosed T2D. Their preadmittance HbA_1c_ was 7.7 ± 1.5%; 47% (189) had HbA_1c_ ≥ 7.4% (SCGC) and were moved to the upper step in the therapeutic algorithm at discharge. Another 15 subjects (3.6% of the cohort) had T2D according to their current HbA_1c_. Ninety-four of the 189 SCGC patients were evaluated 3–6 months later. Their HbA_1c_ before in-hospital-intervention was 8.6 ± 1.2% and 7.5 ± 1.2% at follow-up (*P* < 0.004). Active detection of hyperglycaemia in patients admitted in conventional surgical beds permits the identification of T2D patients with SCGC as well as previously unknown cases. A shift to the upper step in the therapeutic algorithm at discharge improves this control. Hospitalization is an opportunity to break therapeutic inertia.

## 1. Introduction

Maintaining good glycaemic control reduces the risk of microvascular and macrovascular complications associated with type 2 diabetes (T2D) [1, 2]. However, despite a broad armamentarium of effective glucose-lowering therapies, almost half of patients with T2D do not achieve globally recognized blood glucose targets [3, 4]. Between 25 and 40% of patients admitted in conventional hospitalization beds for other reasons have T2D. Hyperglycaemia in hospitalized patients, regardless of the cause, is associated with increased morbidity and mortality and it is known that early diagnosis and treatment improves the general outcomes [5–8].

Therapeutic inertia (TI) is the delay in the onset or intensification of a required treatment [9]. TI exists in a considerable percentage of patients with T2D, being reported in about 50% of cases [10, 11]. Hospitalization for other causes than diabetes could be a good opportunity to detect patients with T2D and poor glycaemic control and thus overcome TI, and, moreover, it may allow detecting unknown cases. Surgical departments of general hospitals usually have a very high prevalence of T2D patients.

The objectives of this prospective study were to set up a programme for active detection and treatment of hyperglycaemic patients during the admission in conventional surgical beds, to identify patients with previously known T2D that have a suboptimal chronic glycaemic control (SCGC) and to evaluate the impact of treatment modifications indicated at the time of hospital discharge on the glycaemic control at midterm.

## 2. Material and Methods

This new intervention programme started in May 2012 in Hospital Germans Trias i Pujol, a tertiary referral hospital affiliated to the Universitat Autònoma of Barcelona, Catalonia, Spain. The Centre has 509 beds, with 353 of them in conventional hospitalization and 150 of those devoted to surgical services. The protocol has been progressively implemented in five surgical hospitalization departments (orthopaedics and traumatology, vascular surgery, general and digestive surgery, neurosurgery, and urology) in the last two years, and a pre- and postintervention assessment were planned in order to evaluate their efficacy. Admitted patients in these departments are noncritically ill ones and hyperglycaemia was defined as premeal blood glucose greater than 140 mg/dL, following the specific recommendations of ADA for management of hyperglycaemia in hospitalized patients [12–14]. Detection of hyperglycaemia in our centre uses an electronic warning message by which a glycaemic threshold of 140 mg/dL is automatically generated from admittance analyses for all the patients of surgical wards and then communicated to the Endocrinology Service by midday. The generated list of patients is then revised by a team of senior and junior residents and by a nurse and supervised by a senior staff member. The electronic clinical histories of these patients and their recent HbA_1c_ levels are evaluated; if there is no <3 months data regarding HbA_1c_, a new measurement is performed with the recent blood sample from the present hospital stay, following the recommendations of the ADA [14]. HbA_1c_ was measured in blood samples with ethylenediaminetetraacetic acid (EDTA) by high-performance liquid chromatographic (HPLC) using a fully automated Adams Menarini HI-AUTO A1c 8160 analyzer manufactured by Arkray (Kyoto, Japan) with an interassay coefficient of variation of 1.8 and 1.5% at HbA_1c_ levels of 4.8 and 9.0%, respectively (reference range: 4–5.8%). This method is a cation exchange HPLC method certified by the NGSP (National Glycohemoglobin Standardization Program) of traceability to the Diabetes Control and Complications Trial Reference Method (DCCT).

Hyperglycaemic patients were categorized into one of the following four groups: (A) controlled T2D: T2D previously known and HbA_1c_ < 7.4%; (B) T2D: previously known with SCGC as defined by a HbA_1c_ ≥ 7.4%; (C) T2D: not previously known as defined by hyperglycaemia > 140 mg/dL and HbA_1c_ ≥ 6.5%; (D) undetermined hyperglycaemic status, as defined by hyperglycaemia > 140 mg/dL and HbA_1c_ < 6.5%.

During hospital stay, all patients were treated following the institutional protocol of control of hyperglycaemia consisting of a basal-bolus insulin therapy designed according to the level of hyperglycaemia and the fasting condition required for the perioperative period. When patients were discharged, those in group (B) (HbA_1c_ ≥ 7.4%) received a reassessment of their nutritional plan and were moved to an upper step of the therapeutic algorithm, following the recommendations by the Spanish societies of endocrinology and diabetes and the EASD/ADA [15, 16]. Patients in group (A) (well controlled diabetic patients) remained under the same treatment as that before their hospital admission. New diagnosis of T2D patients (group (C)) was initiated in diabetes treatment according to the HbA_1c_ level and clinical judgement by the consultant diabetologist team. All patients, regardless of the group, received the recommendation to make a follow-up appointment by their primary care team at 3–6 months of the hospital discharge. All treatments changes were specified in a highlighted manner in the discharge reports. In those patients with SCGC (group (B)) HbA_1c_ was determined again between three and six months after hospital discharge and evaluated according to the usual criteria by their primary care team. We compared these with previous results by consulting the electronic clinical history.

Continuous variables were expressed as mean standard deviations (SD) or median (interquartile range) and categorical variables as frequency and/or percentage. Differences between groups were assessed by the Student's *t*-test or the nonparametric Mann-Whitney *U* test, as appropriated. A *P* value less than 0.05 was considered statistically significant. Categorical variables were compared with *χ* ^2^ test. All statistical analyses were performed using the Statistical Package for Social Science (SPSS, Chicago, IL, USA) for personal computers, version 12.0 (SPSS).

## 3. Results

Four hundred and twelve hyperglycaemic patients were detected during the first 18 months after initiation of the programme. Of the 412 patients, 193 (47%) had an acute illness and the rest (219; 53%) had been planning program admission. The most common reasons for admission in patients with an acute process were bone fractures, mainly femur, arterial peripheral ischemia, cholecystitis, pancreatitis and other abdominal infectious processes, hemorrhagic stroke, and urological infections. The most frequent diagnoses of patients with scheduled hospital admission were chronic degenerative arthropathy, arterial stenosis, lesions that needed to be amputated, neoplasias of the digestive tract and the central nervous system, spinal disc herniation, and tumors of the kidney and prostate. Of the total 412 patients, 357 (86.6%) had previous known T2D (groups A and B) and 145 were women (40.6%), with a mean age of 69.7 ± 10.4 years, T2D evolution of 9 ± 9.5 years, and HbA_1c_ of 7.6% ± 1.4. From the 357 patients with known T2D, 168 (52.9%) were under good control (group (A), HbA_1c_ 6.5% ± 0.5). They had a mean age of 70.4 ± 11 years and a T2D evolution of 6.5 ± 6.1 years. The remaining 189 patients (47%, group (B), HbA_1c_ 8.6% ± 1.3) had a mean age of 69.2 ± 9.8 years and a T2D evolution of 10.9 ± 10.8 years. Patients in group (B) had a longer evolution of the T2D in comparison to those of group (A) and their HbA_1c_ was also higher (*P* < 0.001 for both). Fifteen patients had newly detected T2D (group (C)), corresponding to 3.6% of the total cohort, and in 40 patients the hyperglycaemic status was found in conjunction with an HbA_1c_ < 6.5% (group (D)). Mean age in group (C) was 68.8 ± 12.7 years and in group (D) 60.7 ± 18.3 years.

Patients in group (B) were moved to the upper step of the therapeutic algorithm [15, 16] at the time of hospital discharge. This action included the initiation of insulin therapy in 28 out of 357 patients with known T2D (14.8%). Ninety-four of the 189 patients from those whose therapeutic changes were performed have been assessed 3–6 months after discharge by consulting the primary care electronic clinical history. Their HbA_1c_ improved significantly (preadmission or in-hospital HbA_1c_ 8.6% ± 1.27 and after discharge 7.5% ± 1.25, *P* < 0.004). These data are shown in Table 1. In the remaining 95 patients with SCGC, 45 showed no HbA_1c_ in the clinical history due to a lack of scheduled follow-up visit after discharge and in the other 55 the follow-up visit was still not performed, as the time elapsed from discharge was less than 6 months.

## 4. Discussion

Recently, different diabetes medical societies have made specific recommendations for the care of diabetic patients regarding their glycaemic control when they are in hospital for any cause not specifically related to diabetes. Moreover, there is a general feeling that a certain delay exists in detecting diabetic patients and initiating the specific protocol for hyperglycaemia control while the patient is hospitalized, in particular in those admitted to surgical wards [17]. These scientific societies have proposed to take action against this situation by implementing both active detection and adequate treatment of diabetic patients when they are admitted to the hospital irrespective of the cause of admission [5]. Diabetes is present in a proportion as high as one-third to half of patients in community hospitals, a figure that will grow steadily in the near future in industrialized countries due to the increasing prevalence of diabetes, mostly related to ageing. The possibility to have automatic warnings that indicate the presence of the diabetic condition in a given patient provided from different hospital check points allows gaining time when classifying which patients require more prompt attention. Our tertiary hospital has an electronic clinical history shared with primary care physicians; thus current and past relevant information from a specific patient can travel across the health system on a real time basis.

Our protocol allowed us to detect 357 known T2D patients among those admitted to conventional beds of surgical services. Most of these would probably not have been considered as potential diabetologic consultations by their physicians in charge, as assessed by the comparison of the historical number of calls received from the specific surgical services included in the programme and the number of patients detected by the current programme. These patients are noncritically ill and a very heterogenic group. The challenge of defining the goals of antihyperglycaemic therapy arises from their heterogeneity mainly in relation to the aging process along with their diverse clinical characteristics. As a group, they should be treated as elderly diabetic patients. Therefore, it was considered that patients with HbA_1c_ < 7.4% were controlled [18]. Almost half of the patients detected (47%) had a previous SCGC at the time of hospital admission according to HbA_1c_ values. Some studies conducted in different geographical areas in Spain have confirmed a similar percentage of T2D patients with poor glycaemic control when primary care databases are evaluated [19–21]. These figures may be even higher when complex diabetic patients with active comorbidities and mostly followed at tertiary hospitals are concerned. Overall, the present study demonstrates that a substantial number of patients show a significant—either clinical or statistical—improvement in the glycaemic control at short- and midterm after active detection, evaluation, and modification of the therapeutic programme for every specific case. Consequently, we observed a 1% mean decrease of HbA_1c_, thus reaching the recommended 7.5% HbA_1c_ value for this age group of patients. Additionally, 3.6% of the total cohort corresponded to new cases; thus our programme allowed an early treatment in these particular patients or at least did not further delay the diagnosis of T2D.

Therapeutic inertia (TI) is defined as the situation by which a given patient requiring a next step treatment modality usually with higher complexity does not receive the appropriate treatment. TI seems to be present approximately in one-third to 40% [22, 23] or even more [24, 25] of T2D patients with poor glycaemic control, especially those treated only with lifestyle changes or oral monotherapy, and also in older subjects. Assessing the true prevalence of TI is difficult and it should be noted that the methodology used to obtain these figures is heterogeneous. Moreover, TI is not the same as clinical inertia (CI) which includes not only the responsibility of the physician at the time of escalation in the therapeutic algorithm towards more complex treatment modalities but also the position of the patient, in which he/she voluntarily decides not to follow the therapy proposed by the diabetes team. In this regard, CI requires educational and emotional support, while TI requires medical training and support from expertise. Finally, the evaluation of HbA_1c_ as the indicator of TI should also be refined according to individual goal convenient for every patient, mostly related to concurrent diabetes complications and age. Therefore, a given patient may have a convenient HbA_1c_ 8% value if he/she has major comorbidities and/or is very old and frail. However, this same value is inadequate for younger subjects with no apparent active comorbidities and relatively short duration of the disease.

In a recent multicentre, retrospective study of patients with poorly controlled diabetes and at least one hospitalization [26], less than a quarter received a change in their diabetes therapy upon discharge, and nearly one-third had no subsequent follow-up visit scheduled, suggesting widespread TI. In our cohort, a substantial number of subjects, around a quarter, did not have a primary care scheduled visit 6 months after discharge. This approach of controlling the whole process after discharge by temporal assessment of the shared electronic clinical histories also allows the implementation of rescue actions towards the reinclusion of patients lost in the follow-up by means of phone calls and other ways of contact. The overall approach could, therefore, increase the quality of care for T2D.

The implementation of an active detection programme and treatment of hyperglycaemia in patients admitted in conventional surgical beds, such as the one presented in this study, is, therefore, feasible in the habitual clinical practice and necessary for a substantial proportion of patients. We also demonstrated that the modification of the previous treatment to an upper step in the diabetes therapeutic algorithm together with the personalization of recommendations in patients with type 2 diabetes is able to obtain a significant improvement in the glycaemic control, at least at midterm.

## 5. Conclusions

Admission in a conventional surgical bed for any cause is a clear opportunity for overcoming therapeutic inertia and improving glycaemic control in patients with type 2 diabetes. We, therefore, propose the implementation of an active detection and treatment programme of hyperglycaemia, as we describe here, in all community and tertiary hospitals.

## Figures and Tables

**Table 1:** Data of 94 out of 189 patients (group (B)) whose therapeutic changes were performed and were reassessed 3–6 months after discharge.

*N*	Women %	Age (years)	DM evolution (years)	HbA_1c_ PRE (%)	HbA_1c_ POST (%)
94	37 (39.4)	68.94 ± 9.89	12.44 ± 11.88	8.66 ± 1.27	7.50 ± 1.25^*^

^*^ *P* < 0.004.

*N*: number of patients.

DM: diabetes mellitus.

HbA_1c_ PRE: preintervention.

HbA_1c_ POST: postintervention.
